# On Combining Convolutional Autoencoders and Support Vector Machines for Fault Detection in Industrial Textures

**DOI:** 10.3390/s21103339

**Published:** 2021-05-11

**Authors:** Alberto Tellaeche Iglesias, Miguel Ángel Campos Anaya, Gonzalo Pajares Martinsanz, Iker Pastor-López

**Affiliations:** 1 Computer Science, Electronics and Communication Technologies Department, University of Deusto, Avenida de las Universidades 24, 48007 Bilbao, Spain; camposanaya.miguel@opendeusto.es (M.Á.C.A.); iker.pastor@deusto.es (I.P.-L.); 2 Software Engineering and Artificial Intelligence Department, Complutense University of Madrid, Calle del Prof, José García Santesmases, 9, 28040 Madrid, Spain; pajares@ucm.es

**Keywords:** image sensors, texture inspection, autoencoder, SVM, hybridization

## Abstract

Defects in textured materials present a great variability, usually requiring ad-hoc solutions for each specific case. This research work proposes a solution that combines two machine learning-based approaches, convolutional autoencoders, CA; one class support vector machines, SVM. Both methods are trained using only defect free textured images for each type of analyzed texture, labeling the samples for the SVMs in an automatic way. This work is based on two image processing streams using image sensors: (1) the CA first processes the incoming image from the input to the output, producing a reconstructed image, from which a measurement of correct or defective image is obtained; (2) the second process uses the latent layer information as input to the SVM to produce a measurement of classification. Both measurements are effectively combined, making an additional research contribution. The results obtained achieve a percentage of success of 92% on average, outperforming results of previous works.

## 1. Introduction

Texture identification in images, from different materials, is a key problem in computer vision. A texture can be defined as a basic pattern (molecular) arrangement that repeats itself in a structured way on object surfaces creating recognizable patterns in the images. Therefore, each pattern defines inherent and specific texture patterns depending on its molecular structure, allowing the discrimination of different materials through the images they produce. Typical materials with textures are, for example, wood fibers, granite plastic or metal grids, textiles, etc.

In industrial production and manufacturing processes, involving textured materials, defects in the final product becomes a serious handicap, generating significant economic losses due to drop in product quality. In this regard, the design of mechanisms to address this problem, the identification of defects, expressed as distortions, ruptures or even disappearance of patterns, is critical. These defects can be captured with images, and the application of automatic computer vision-based techniques appear as promising approaches. An important problem in defect detection, based on computer vision approaches, is that defects can appear under whimsical shapes with irregular and unstructured patterns and without specific localizations. Therefore, recently there has been a great interest towards the development of algorithms that focus on the detection of “outliers” in the texture under inspection, or what is the same, the accomplishment of the classification of a class, in which any defect that does not fit to the learned patterns, for a specific texture, will be classified as not belonging to the basic class of the correct texture.

This research work proposes a solution, for the defect detection in textures, that combines two machine learning-based approaches as classifiers: (a) convolutional autoencoders (CA) in deep learning, and (b) one class support vector machines (SVM). Both methods are trained by only using fault (defect) free textured images for each type of texture, allowing the automatic labeling of samples for the SVMs. With this proposal, only correct images are used for training both classifiers, achieving an important advantage against existing methods that avoids the need to select defective textures, which are always difficult to define due to the capricious appearance of defects.

The convolutional autoencoder is used for two fundamental operations, firstly for obtaining the reconstruction error from a given textured image to identify inherent defects present in surfaces and embedded in the image, and secondly, for the compression of the texture information in its latent layer. This compressed information will later be used to train the SVM.

It is well known that autoencoders have been used, for a long time, for classification tasks [1] because of their potential in reducing data dimensionality for feature extraction [2]. The main advantage of data dimensionality reduction is the elimination of redundant information and noise in the incoming data, very common in the unstructured textures addressed in this project. This justifies the use of autoencoders in our proposal.

In this regard, the main contribution of this research work is based on two image processing streams stablished through the CA: (1) the first processes the incoming image from the input to the output, producing a reconstructed image, from which a measurement of correct or defective image is obtained; (2) the second processes the same image through the coder, just until the latent layer, where it is compressed and mapped as a latent vector, which is the input to the SVM to produce a measurement of classification. The use of only the autoencoder is justified under the assumption that early convolutional layers extract general, low-level features such as patterns that characterize the textures. Both measurements are conveniently combined, making an additional contribution, i.e., a hybrid approach. As explained above, only defect free samples will be used for training both algorithms, and hence, the SVM training labels automatically all the samples in its training stage as belonging to the defect free class.

### 1.1. Methods Guided by Structured Patterns

In the study of textures with the aim of detecting defects, it is important that the proposed solution is invariant to the orientation and scaling of the texture [3] to ensure the robustness of the results.

Within classical methods of texture analysis, three different categories of methods can be distinguished depending on the approach used: statistical, structural, and model-based. Statistical methods were the first developed. In 1981, Davis [4] defined a tool he called a polarogram, which allows to obtain invariant characteristics for textures. The polarogram is defined as a graph in polar coordinates that represents statistics of a texture with respect to its orientation. Results for textures with 16 different orientations are reported.

Like Davis, Mayorga and Ludeman [5] also used polar frames, thus achieving invariance in rotation, but unlike Davis, they used texture edge data by deriving them in certain directions. This method presented problems of variability according to the method used to obtain edges of the texture, with high dependency on the edges defining the texture.

In the study by Pietikainen [6], the texture image is presented based on the symmetric autocorrelation of the center, the local binary pattern and the gray density. The characteristics obtained were mostly invariant to rotation. The main problem of this method is that it is only efficient for highly ordered textures, but not in unstructured textures where results are ineffective.

Local binary patterns (LBP) [7] is one of the most classical methods within the classification of textures in computer vision. A binary histogram is obtained by considering the neighborhood of the pixel under study, obtaining a characteristic vector after normalization. This method is combined with the Histrogram of Oriented Gradients (HOG) [8] to increase its performance. The invariant moments of an image allow also the recognition of their patterns [9]. In unstructured texture patterns these approaches are not appropriate because no HOG patterns can be derived.

Of all existing moments, Teh and Chin [10] show in their study that Zernike’s moments are the best results when applied to textures. The orthogonality of these moments gives them an invariance against rotation. Wang and Healey [11] used these moments in multispectral functions and established relationships to obtain invariance under different illumination conditions. This is not the specific problem for defect detection in textures.

Another alternative method applied is harmonic expansion. This method achieves the characteristics of textures by decomposing their harmonic components into their polar form, and their subsequent projection, obtaining invariant coefficients to the rotation with which the texture pattern can be defined. The implementation carried out in 1985 by Alapati and Sanderson [12] includes these types of characteristics, but the method developed was only rotationally invariant, i.e., not suitable for defects, that does not include rotational patterns.

In 1992, Tsatsanis and Giannakis [13] used high level descriptors obtained through cumulative and multiple correlation. A major disadvantage of this method is its high computational cost and that the high order descriptor may have lost too much information in order to correct texture discrimination.

Another group of alternatives is model-based methods, where the texture image is modeled as a probabilistic distribution or a linear combination of a set of basic functions. The coefficients of these models are used to characterize the textured image. The key issue of these methods is how to estimate the coefficients of these models and how to choose the correct model for the selected texture.

Bovik et al. [14] used Gabor filters, obtaining results of 90% accuracy, combining these filter sets with elementary transformations of invariant textures. Studies on Gabor filters such as the one carried out by Randen [15] indicate that this type of method outperforms others in terms of complexity and error rate. Texture defects do not display patterns as defined by Gabor filters.

Cohen [16] used Markov models to model textures. Other implementations with this model were carried out by Chen and Kundu [17] where texture descriptors were obtained by using multichannel sub-band decomposition and the hidden Markov.

Another model-based method widely used in the literature is the SAR one. This model is rotationally invariant, and Kashyap and Khotanzad [18] developed an autoregressive circular model (CSAR) considering circular neighborhoods. Mao and Jain [19] improved the previous approach with a model they call (RISAR), in which the weighted grey values are separated into several circles; so that, after applying the rotation they are approximately equal. However, some problems arise, such as how to choose an appropriate size of neighborhood or how to select a window size in which the texture is considered homogeneous.

Finally, in structural methods, the full texture pattern is divided into texture elements arranged according to placement rules, so that structural properties can be derived.

Goyal [20] used the perimeter and compactness of the basic structural elements of a given texture assuming invariance. He converted the original histogram of the image into an invariant one, taking into account the number of structural elements existing in the case under study.

Another algorithm based on structural methods is morphological decomposition. Lam and Lin [21] use invariant iterative morphological decomposition (IMD) for classification. By means of this method, the texture is decomposed into a set of composite images and some statistical characteristics (mean, variance, normalized variance and gradient) are obtained for each component.

Finally, Eichmann and Kasparis [22] base their analysis on the Hough transform. They consider the rows as the points in the plane of the transform and since the Hough transform is performed on the binary image, they use the Radon transform to implement the Hough transform for the non-binary textures. This method can, therefore, be understood as a topological texture descriptor.

As mentioned in the title of this section, all the above methods are based on structured patterns defining the texture. Thus, when trying to identify defects, characterized by unstructured textured patterns without geometric relations, as expressed before, the above techniques are difficult to apply. This would require establishing the structural and geometric relationships between correct and defective patterns to study the differences, which is inappropriate. Consequently, the most desirable option is to apply global strategies without the need to define specific strategies for each type of textured pattern, and this is what can be achieved with our proposal based on autoencoders.

### 1.2. Deep Learning Algorithms for Anomalies Based on Texture Analysis

In order to evaluate the performance of deep learning-based approaches, several datasets are available, such as MNIST [23], ImageNet [24], COCO [25], PASCAL VOC [26] or CIFAR 10 [27]. When trying to detect anomalies, there are two common approaches using any of these datasets as the basis. The first one consists in labeling some of the classes present in the dataset as anomalies, so that the proposed method is trained with the classes labeled as normal and its detection capacity is evaluated against the anomalies [28,29,30]. The second alternative is to extend a chosen dataset with images containing anomalies, and then train the method with the aim to work on anomaly detection [31]. The main drawback of these approaches is the definition of images with anomalies which are to be supplied for training.

Within deep learning, different methods have been proposed for texture anomaly detection [32], but two of them are mainly used: antagonistic generative networks (AGN) and convolutional autoencoders.

Schlegl [31] proposed to model the training data using an AGN model trained only with defect-free images. These networks work with a generator and a discriminator. In this way, a latent sample that reproduces the input image is sought, achieving to deceive the discriminator. The anomaly present in the texture is obtained by comparing the input image and the generated image pixel by pixel. This approach is close to the one proposed in this paper with regard to image processing until reconstruction, but this method does not consider the identification of anomalies under the assumption that defects produce high variability in the intensities of the images in the early layers in the network, diluting this effect across the AGN model.

CA [32] are also used to detect anomalies. Generally, the anomaly is detected by considering the reconstruction error obtained by the network when it tries to reconstruct an anomaly not previously trained. To obtain information about the reconstruction error at pixel level, it is necessary to go through the image pixel by pixel. Bergmann [33] points out the disadvantages of this way of comparing the image with the original and proposes incorporating the spatial information of the local regions using structural similarity [34] with the aim of improving the segmentation results. There are also several extensions of CA, such as the variational autoencoders [35] that have been used by Baur et al. [36] for the segmentation of brain MRI anomalies. In this work, Baur et al. use different CA architectures to reconstruct the original brain images used in their study, concluding that these architectures with dense bottlenecks cannot reconstruct these types of images with the required efficiency, not being of practical use for anomaly detection. This leads us to think that the same will happen in our case for defect detection.

In [37], Minhas and Zelek also use autoencoders to create a semisupervised algorithm for anomaly detection, focusing the study on two different datasets, the first being a synthetic one, and the second centered in railway images. They conclude that, while the synthetic dataset performs well, the real dataset presents noisy results. In addition, this approach has been applied to the detection of only one type of defect in the dataset, not being applicable to our case, which presents five different types of defects for five different textures.

Another example of practical use of convolutional neural networks for anomaly detection can be found in the work of Staar et al. [38]. In this case, they use a specific network architecture called triplet network to detect surface defects. The main drawback of this work is that it is a supervised method that needs precise labeling of defects in the dataset to provide effective results. This requirement is not easily fulfilled in industrial products, because the defects considered in our approach contain unpredictable and fanciful forms.

Finally, Maldonado et al. [39] focused the work on the capacity of transferring trained parameters in an unsupervised domain to other problems where the dataset is supervised. The considerations of this paper are valuable in processes of fault detection, where in many cases the error cannot be categorized correctly due to the lack of samples with defects available.

In short, when compared to these previous works, our proposal uses a real industrial dataset, with up to five different textures coming from many other products that allows us to design a procedure applicable to a great variety of textures for inspection problems. Additionally, we do not need any type of labeled dataset, and by combining two different approaches for anomaly detection like CA and one class SVM, the proposed solution outperforms the results obtained using CA only, which are aligned with the approaches mentioned above.

Erfani et al. [40] deal with anomaly detection by combining a deep belief network approach that extracts features, which are used as inputs for one class SVM classifier. The features are obtained in the compressed latent space. This is a combined approach where anomalies are detected by the SVM and the network provides the high dimensionality in the input space. Our proposal goes further, in the sense that it also exploits this idea but also uses the network, in this case the autoencoder, to classify textures for anomalies detection and free of defects. Then, it combines both results for a final decision, in what we have called hybridization, making a major difference from such work. Additionally, the experiments reported in this work indicate that the performance of nonlinear kernels in SVM can be replaced by linear ones because of the generation of high dimensionality vectors. We also exploit this fact verifying the same behavior in our experiments. This justifies the use of the linear kernel in the proposed SVM.

Anomaly detection in images has also been considered in Beggel et al. [41]. They use the latent space in an autoencoder to model a likelihood for discriminating textures with and without anomalies, based on what they call density estimation, i.e., determining the degree of occurrence of data in the vector space. High concentrations would indicate fault-free textures and deviations from these concentrations would imply anomalies. This work, together with the previous ones, confirms the importance of the use of latent space in the detection of anomalies, which is also exploited in our approach.

This paper is organized as follows: Section 2 presents in a concise manner the main contributions of this paper over the state-of-the-art. Section 3 presents the theoretical design of the solution proposed, focusing on the design principles of the autoencoder and the SVMs, also establishing the hybridization method to optimally combine the complementary outputs of both approaches. Section 4 presents the public dataset used for performance evaluation of the algorithms and the results obtained, giving detail about the different classes presented in it, type of errors and number of samples of each class and errors. This section also gives detail about the training procedures of both algorithms and results obtained for the case of use presented in this research work. Finally, in Section 5 conclusions extracted from this work are summarized, justifying the suitability of the solution proposed among other alternatives.

## 2. Main Contributions

This paper presents a novel research work that fuses the classification information of two different approaches, CA and SVM, to effectively detect defects in different textures commonly present in industrial applications. More precisely, the following main contributions can be highlighted:

▪ A compact CA architecture was used in a very promising way to extract valuable information on its output used for defect detection in textures, the reconstruction error and the compressed information in the latent layer.
▪ The use of SVMs to model the compressed high dimensionality information extracted from the latent layer of the CA. The use of the latent vector was reported as an efficient approach as indicated in Section 1.2, i.e., aligned with our proposal. The latent vector can be considered as the input for different classification algorithms, and we exploited this fact by using SVM. We verified that SVM outperforms other existing classical strategies such as the probabilistic parametric Bayesian approach [1] and the fuzzy clustering [1,2], which was used for benchmark. This, together with the its one-class definition, is another powerful reason for using SVM. The robust unknown defect detection approach combines complementary outputs of CA and SVMs.
▪ The proposed approach outperforms CA and SVM when they are applied separately. This results in an implicit outperformance of the existing strategies described in the state-of-the-art.

## 3. Methods: Fault Detection Process in Textures

**Figure 1:**
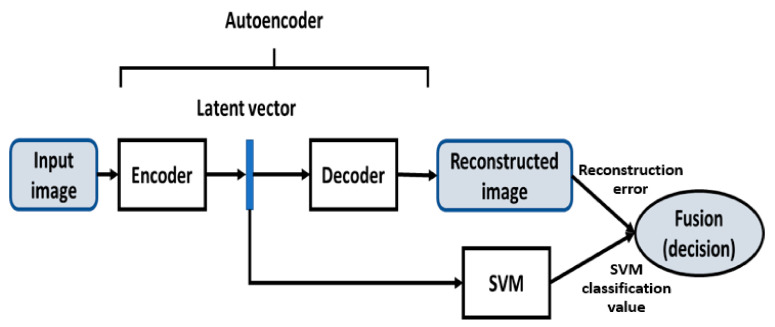
Main schema of the combined solution.

As stated above, the proposed approach combines a CA and SVM for effective texture defect detection. This section deals with the formal design of both methods and the subsequent hybridization of their outputs to create a robust detection method. Figure 1 displays the architecture of the proposed model, where the different modules are integrated for making a final decision at the last phase of fusion. The input image evolves through the autoencoder until the latent vector is obtained. Then, the processed image progresses towards the part of the decoder until the output, where the reconstructed image is compared with the input one, obtaining a first measure relative to the content of the texture. The latent vector, which contains details from the input image, when layered shallowly, is fed to the SVM for a second texture measure. Both measurements are combined to make the final decision on the type of texture. This scheme is valid for both, the training phase, where only defect-free images are used, and for the decision phase.

### 3.1. Convolutional Autoencoder Design

In the design of the autoencoder, a balance was considered between (a) the development of a compact architecture that offers a quick and effective response and (b) with enough learning capacity to effectively generalize the underlying structure in the textures under study. Its basic architecture was created taking into account the good results that the CNN architectures Darknet-19 [42] and Darknet-53 [43] have offered when used as backbone CNN for the YOLO object detector. Following their basic structure, we adapted it to create an effective CA. Optimizers and learning parameters were also studied taking into account the design information present in these references.

**Figure 2:**
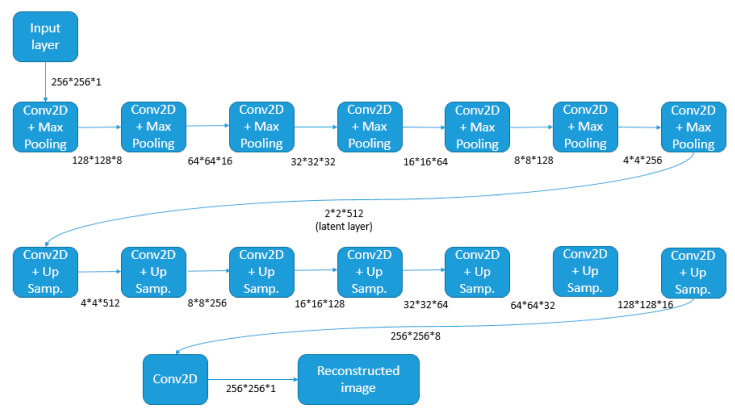
Main structure of the proposed convolutional autoencoder.

A convolutional autoencoder was chosen due to the advantages of deep learning in the automatic extraction of features. In general, an autoencoder is defined mathematically as:

(1) $$\phi :X\to F\;\psi :F\to X\;\phi ,\psi =argmin_{\phi ,\psi }||X-\left(\psi \cdot \phi \right)X{||}^{2}$$

where *ϕ* is the encoder and *ψ* is the decoder. *F* represents the latent variables layer, where the autoencoder compresses the underlying information from the input that allows the decoder to reconstruct the output. Figure 2 shows the basic architecture of the autoencoder used in this proposal. The encoder consists of seven layers grouped as convolutional (Conv2D) layers with Max-pooling. The decoder contains seven Up-sampling Conv2D layers plus the final Conv2D. The different sizes of feature maps after each layer are also displayed, including the latent vector with size 2 × 2 × 512.

The autoencoder design decisions were carried out empirically throughout the development of the experiment and are justified below.

The total number of trainable parameters of the autoencoder is 5.5 × 10⁶. This number, directly derived from the proposed architecture, has proven its efficiency for the reliable detection of defects in the five basic texture types presented in the previous section.

The color images in the original dataset were transformed to grayscale images, so that the autoencoder learns the underlying texture patterns independently of their color. The size of each input image to the autoencoder is then 256 × 256 × 1 pixels.

The size of the input image is justified by looking at the average sizes of the different texture defects present in the validation images of the dataset. By selecting the most restrictive defects, that is, the smallest ones in number of pixels, and assuming that the defect must be present in at least one portion that represents the 20% of the input image to be detected in a robust way. This portion is the input image obtained from the original full image.

The proposed autoencoder structure is known as undercomplete, because the dimension of the latent layer is much smaller than the input layer. By using this type of architecture, the autoencoder is forced to learn the most salient features of the input images. Ideally, this layer has to maintain a small dimension when compared to the input data size; otherwise, if it has too much capacity, the autoencoder performs a copy task, without extracting useful information from the input dataset.

In our case, the latent layer of the autoencoder has a dimension of 512 × 2 × 2, or 2048 neurons. This result becomes a 32:1 compression ratio, achieving a good balance between texture representation and information compression. As mentioned before, the compressed information of this latent layer will be used as input vector for the SVM algorithm.

**Figure 3:**
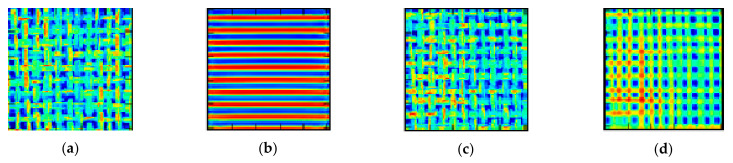
Reconstruction error for a latent layer with 1024 neurons (left, images (**a**,**b**)) and with 2048 neurons (right, images (**c**,**d**)).

This size of the latent layer was established empirically by varying its size and attending to the mean reconstruction error obtained for the different textures. For a size of the latent layer smaller than 2048 neurons (64, 256, 512 and 1024), the CA does not have enough generalization capacity to reconstruct the textures effectively. This can be easily observed in Figure 3, in (a) and (c) original images and in (b) and (d) their corresponding reconstructions with a latent layer of 1024 and 2048 neurons, respectively.

Consequently, as displayed in the results above, it is obvious that for our task, a latent layer of 2048 neurons, i.e., a vector of such dimension, provides an effective reconstruction while maintaining good compression capabilities.

Regarding the selection of the optimizer in the CA, initially two main optimizers were considered for training [44]: ADAM and RMSprop. The learning rate was set to 0.001 for both optimizers and β_1_ = 0.9 and β_2_ = 0.999. These two optimizers were preferably selected over SGD (stochastic gradient descent) optimizer, after taking into consideration tests and results presented in [44]. According to this research work and the typology of the data present in the dataset used, ADAM and RMSprop optimizers offer better training results without the need of taking special care in the tuning of the learning rates.

In order to evaluate the capacity of autoencoder learning with both optimizers, the autoencoder reconstruction error for a given image is defined as:

(2) $$E=\frac{\sum_{i=0}^{m}\sum_{j=0}^{n}\left(\left|a\left(i,j\right)-b\left(i,j\right)\right|\right)}{m*n}*100$$

As derived from the expression (2), a lower percentage of reconstruction error implies a better image reconstruction response to an input texture by the autoencoder. This reconstruction error definition is used to determine the best optimizer to be used in the proposed approach. With such purpose, a subset of images containing five different textures were selected, and the autoencoder was trained with both optimizers, computing the average error in terms of percentage and obtained during the learning phase. Table 1 displays the reconstruction errors obtained for each optimizer, for the number of images with correct textures, i.e., without anomalies. The RMSprop optimizer achieves a lower average reconstruction error, so it was chosen as the optimizer with the best performance proposed autoencoder model.

### 3.2. Design and Training of the One Class SVMs

The original algorithm of the support vector machines is a supervised algorithm, initially created for a two-class separation of the input data. According to different existing works, SVM has proven to be particularly effective in classification problems where the samples are not easily separable and present a high dimensionality [45,46,47].

The main idea of this algorithm is based on the fact that, given a dataset of vectors labeled as belonging to two different classes,

$$\Omega =\left\{\left(x_{1},y_{1}\right),\left(x_{2},y_{2}\right),\ldots ,\left(x_{n},y_{n}\right)\right\};\;\;x_{i}\epsilon {\mathbb{R}}^{d},$$

and being the class label $y_{i}\in \left\{-1,1\right\}$. If these data are not easily separable, they can be separated by means of a hyper-plane, mapping the data to a different space.

For the mapping of data vectors to the higher dimensional feature space, transformation functions or kernels are used, which may or may not be linear (linear, polynomial, radial base function or sigmoid).

In this work, and as previously explained, only one class of training samples is available, so the one class SVM algorithm proposed by Schölkopf [48] was used. The data vectors used for training are directly extracted from the latent layer of the convolutional autoencoder, which represents an incoming image of 256 × 256 pixels in a coded vector of 2048 components, sized 512 × 2 × 2. The number of samples for the learning process of each texture is therefore the same as the textured images free of defects used by the autoencoder, collected in Table 2.

Since the input data is high-dimensional, a linear kernel was used, as proposed in [47]. SVM decision function returns +1 for vectors belonging to the trained class returning −1 in the case of outliers. Equation (3) shows the quadratic programming minimization function, and the decision function using Lagrange techniques for its solution.

(3) $$\underset{w,\delta_{i},\rho }{\min}\frac{1}{2}||w{||}^{2}+\frac{1}{vn}\overset{n}{\underset{i=1}{\sum }}\delta_{i}-\rho \;f\left(x\right)=sgn\left(\left(\omega \cdot \phi \left(x_{i}\right)\right)-\rho \right)=sgn(\overset{n}{\underset{i=1}{\sum }}\alpha_{i}K\left(x,x_{i}\right)-\rho )$$

where *w* is the director vector of the class separation hyperplane, *ρ* the independent term of the hyperplane, *δ_i_* are the slack variables to allow some data points to lie within the margin. $\emptyset \left(x_{i}\right)$ is the kernel used, which is linear in this approach. The parameter *v* in Schölkopf’s one class SVMs is the one that characterizes the proposed solution during the training stage. Specifically, it establishes an upper bound to the fraction of outliers, and as a lower bound to the number of training samples used as support vectors. In our case, as the algorithm is trained with only vectors obtained from textures without defects and a precise adjustment is required to achieve the best performance, *v* was set to 0.9, since we are interested in a precise learning of textures free of defects, as indicated in [48]. This parameter is common for all type of textures, and by using a high value, a generalization of texture information is avoided.

### 3.3. Output Hybridization of Both Algorithms

One of the main contributions presented in this research work is the hybridization of the results obtained by the convolutional autoencoder and by the vector machine support for robust texture defect detection.

The classification output from SVM are two discrete values [+1, −1], where +1 indicates that the texture is correct, and −1 that a defect has occurred.

Therefore, in order to hybridize results obtained from both classifiers, it is necessary to map the result provided by the autoencoder as a reconstruction error to a range of [+1, −1]. The following transformation function is established for this purpose:

(4) $$y=-\tanh\left(m\left(x-\bar{x}\right)\right)\;m=\frac{2}{a-b}$$

All images used for testing, with and free of texture defects, are processed by the CA. For each image a reconstruction error is obtained according to Equation (4), and considering the defective ones, the average value (a) is computed. Similarly, the average value (b) is obtained for the correct ones. Finally, $\bar{x}$ is the mean value between a and b. By using a and b values to define m and $\bar{x}$, the value of the reconstruction error is not underestimated, and a correct mapping of its value is obtained in the range of [+1, −1].

Therefore, according to the above, the reconstruction error obtained as a percentage of the original image by the autoencoder is also mapped to the range [+1, −1] to be fused with the values provided by SVM in the same range. Values close to +1 mean that the reconstructed texture has minimum error with respect the input, and values close to −1 indicate that the texture is indeed wrong for that image, i.e., maximum error.

Finally, the hybridization method proposed for the final decision making (z) is formalized in equation 5, depending on the sign of the decision outputs of the autoencoder (x) and SVMs (y). If the sign of both different outputs is the same, this indicates that the sample is classified in the same class by both algorithms. By multiplying both outputs in the range of [+1, −1] an updated confidence value is obtained. In the second case, where a mismatch in the decision exists, the mean value of both classification values is calculated. This mean value will classify the sample taking into account the bigger value of both outputs, but with a minimized confidence in the final decision

(5) $$z=\left\{\begin{array}{c} x\cdot y\;if\;sgn\left(x\right)=sgn\left(y\right)\\ mean\left(x,y\right)\;if\;sgn\left(x\right)\neq sgn\left(y\right) \end{array}\right.$$

## 4. Comparative Analysis and Performance Evaluation

### 4.1. Database for Fault Detection in Textures

For comparative purposes, the Anomaly Detection Dataset, published by the German company MvTec GmbH [49] was identified as the most suitable for testing.

The dataset consists of 15 different classes containing different anomalies, and five of these classes correspond to basic textures that can be found in different industrial production processes, such as carpet, grid, leather, tile and wood.

**Figure 4:**
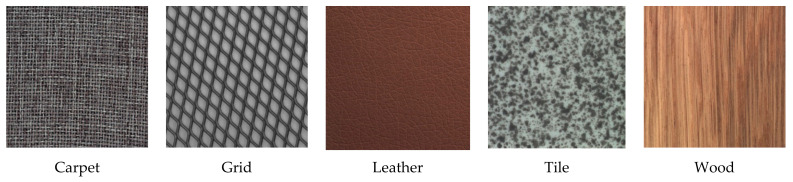
Basic textures used in this research work.

Figure 4 shows examples of these five basic textures, which will be used in this work.

The images in the dataset, for these basic textures, contain a resolution of 1024 × 1024 × 3 pixels, except in the case of granite, which are 840 × 840 × 3. The number of existing fault-free images for each texture is displayed in Table 2. 

This dataset also provides images of defect types in these textures, but in a much lower quantity, similar to what occurs in industrial environments, where production defects are unusual, and therefore there is little data on tagged defects. Five different types of defects are provided for each basic texture. Table 3 indicates the number of images available for each texture and each anomaly.

Table 4 presents visual information on the characteristics of these defects.

This dataset presents interesting characteristics for its direct application during the training process. These are:

▪ The number of images available for each texture is low, besides being of high resolution, resembling the situations encountered in real industrial setups;
▪ The anomalies to be detected are of different shapes and sizes, some of them representing a very small percentage of surface within the whole image, representing a great variability of defects.

In our solution, we designed the following strategy. The original images are partitioned into subimages of smaller dimension, so that a much larger number of lower resolution images are available for each texture.

To establish the subdivision criteria, the tagged defect images available in the dataset were processed, obtaining the bounding box delimiting defect at each image. These data were used to compute the average area (in total number of pixels) for each texture with the results displayed in Table 5.

The most restrictive defect size is, therefore, the one given in the grid texture, with 3173 pix on average. It is assumed for the present work to outline the detection of a smaller defect, established to 2500 pix, that if assumed square, it is equivalent to a minimum detectable defect of 50 × 50 pixel.

As a final design decision, it was established that the size of the minimum detectable defect is not negligible compared to the total size of the input image to be processed. Assuming a 20% occupation of the image surface by the defect, an image size of 250 × 250 × 3 pixel is obtained, finally approximating it to 256 × 256 × 3 pixels. Finally, to the training images present in the dataset, a scaling was applied so that the resolution was uniform for all the textures, obtaining images with resolution of 1280 × 1280 × 3 pixels for the five textures.

**Figure 5:**
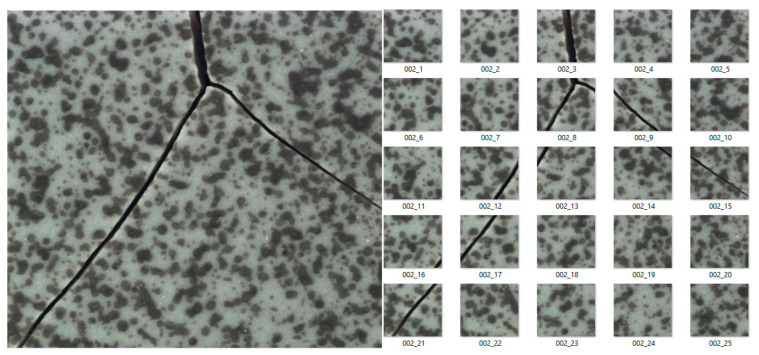
Subdivision of images for the tile texture.

**Figure 6:**
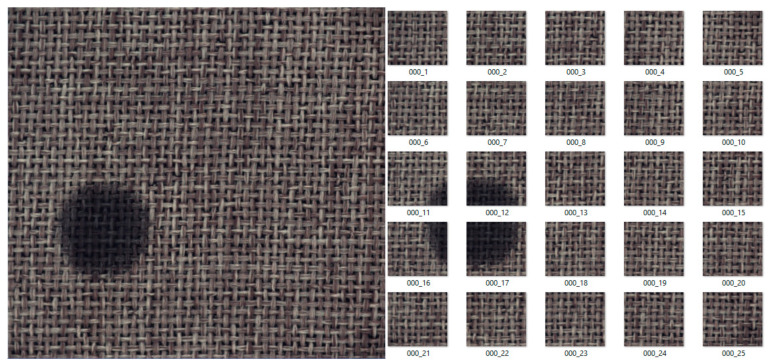
Subdivision of images for the carpet texture.

Applying the subdivision of images explained above, 25 training images were obtained for each input image in the dataset, substantially increasing the images available for the training of the proposed algorithm. Figure 5 and Figure 6 present an example of this subdivision for tile and carpet textures.

Taking into account this subdivision of images, Table 6 displays the number of final training images for each of the five available textures, all with a resolution of 256 × 256 pixels.

As it can be observed, there is now a more adequate number of images without defects for the network training process. In the case of defects, the number of images with defects per texture is somehow unbalanced, and their number is scarce for training purposes, which justifies the detection approach outlined in this paper, based solely on learning correct images for subsequent detection of anomalies.

### 4.2. Training of the Convolutional Autoencoder

The following table displays the distribution of the images of the processed dataset for the training and validation stages of the autoencoder. As mentioned before, the autoencoder is trained only with fault free images; 70% of these images available for each texture will be used for training and the remaining 30% for testing. This dataset split is presented in Table 7.

As established in Section 3.1, a learning rate of 0.001 and *ρ* = 0.9 was established for the RMSprop optimizer, training during 60 epochs with a batch size of 12.

**Figure 7:**
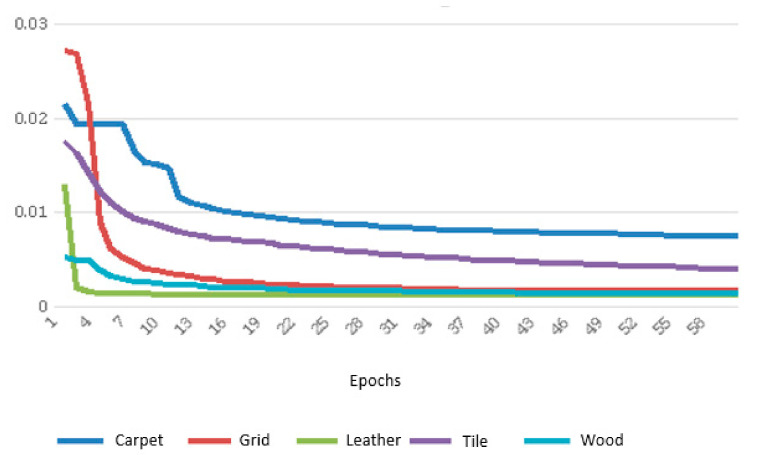
Evolution of the training loss.

In Figure 7, the evolution of the training loss can be seen for each texture under these training conditions.

The training loss reaches values below 0.01 for all textures, which is an acceptable value in this context. However, the learning for some textures, such as leather, wood or grid, is more efficient than for granite or carpet textures.

### 4.3. Results of Convolutional Autoencoder in Texture Reconstruction and Fault Detection

**Figure 8:**
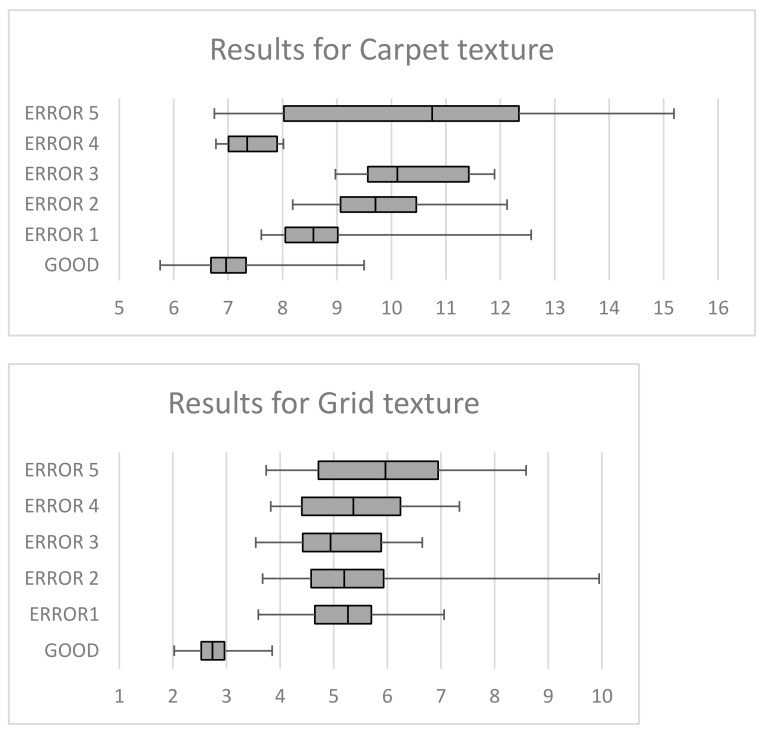

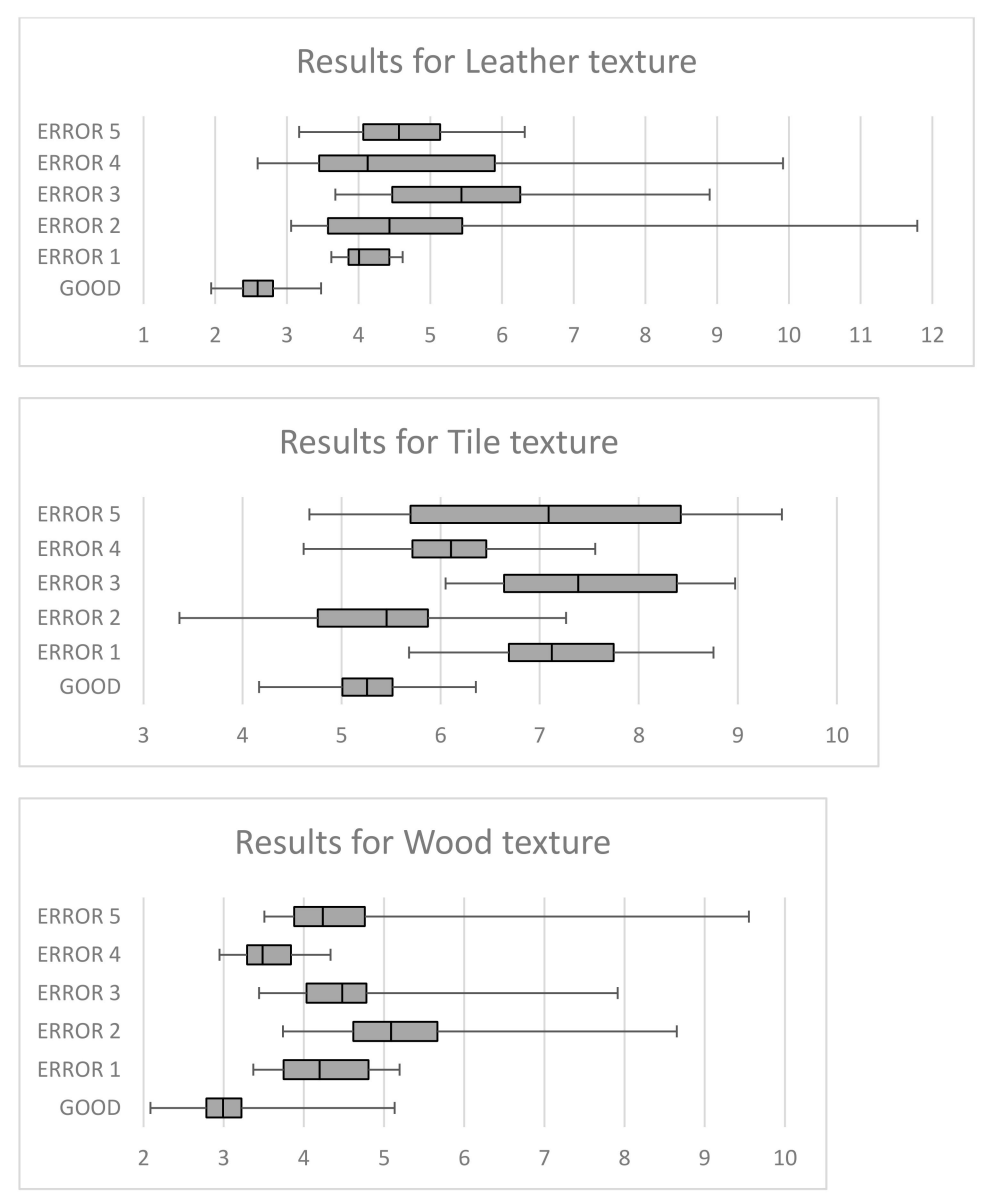
Reconstruction errors for the different textures.

The following graphs in Figure 8 show the reconstruction error obtained by the autoencoder, for each of the different textures, representing the average of the reconstruction error for the textures without defect and for the five types of defects identified for each of them. For each texture and its good and erroneous samples, the minimum, maximum and mean are represented. The gray box represents the quartile of samples below the mean value, while the green one represents the quartile just above this value.

It is important to note that in all the different textures analyzed, the average reconstruction error of the textures free of defects is always lower than the reconstruction error obtained for the images with defects, which shows that the autoencoder works correctly, presenting problems of reconstruction in the images of textures that have defects. Table 8 summarizes the reconstruction error data presented in the graphs above.

For each of the test images, the reconstruction error was calculated, and then its value was transformed according to Equation (6). The negative values are, therefore, images labeled with defects, and the positive values are correct textures.

The following values were defined to evaluate the autoencoder’s ability to detect defects in textures:

▪ True Positive (TP): A texture with a defect is detected correctly.
▪ True Negative (TN): A texture without defects is detected correctly.
▪ False Positive (FP): A texture without defects is labeled as having errors.
▪ False Negative (FN): A texture with defects is labeled as correct.

With these defined values, the *CCP* (correct classification percentage) metric was applied, defined as follows:

(6) $$CCP=\frac{TP+TN}{TP+FP+TN+FN}$$

The following table establishes the *CCP* for each of the textures and defects to be detected, presenting in the last column the average *CCP* for error detection in each case.

As can be seen in Table 9, the CA used independently for defect detection in the proposed textures offers a mean defect detection performance of above 75% in all cases. However, there is some variability in the detection performance of different defects within each texture. As an example, for the carpet texture, defects of types 2 and 3 are detected in more than 90% of the cases, while defect of type 4 for this texture offers a poorer detection performance, slightly above 57%. This variability is the principal weakness of the CA working alone for defect detection in textures. This problem will be overcome with the hybridization of the CA with the SVMs.

### 4.4. Training for One Class SVM

As explained above, the training vectors for the SVM algorithm are derived from the latent layer in the autoencoder for the testing images processed by the autoencoder without defects. That is, once the trained autoencoder is available for a given texture, the latent vector of each defect-free testing image is stored, each vector contains 2048 components the training of the SVM algorithm. The number of defect-free training vectors for each of the textures, therefore, corresponds to the number of defect-free test images presented in Table 7.

As explained in Section 3.2 above, in the design of the SVM algorithm, ν was set to 0.9, and a 5-fold cross validation approach was used for training.

### 4.5. Defect Detection Using SVM

For the evaluation of results, the same metric used for the convolutional autoencoder, the CCP, was also used with the SVM approach. Table 10 shows the results obtained.

These values suggest a very accurate detection of defects present in the textures with a possible overfitting problem, due to the high value of ν, which implies a selection of a high number of support vectors in the training stage. The hybridization with the CA will prevent this known overfitting problem occurring.

### 4.6. Results after Hybridization

To obtain a greater flexibility in the detection of defects of variable characteristics, to prevent overfitting problems and to compensate for the strengths and weaknesses of both detection methods, the hybridization of both algorithms is performed according to the method proposed in Section 3.3 to obtain the final result of texture defect classification.

The results shown in Table 11 correspond to the CCP values computed on the hybrid decision output of both classifiers.

From results it can be inferred that the hybridization outperforms the poor classification results obtained by the autoencoder for certain types of defects, and on the other hand it manages to obtain a more robust defect detection at global level for all textures and for all types of defects.

As previously exposed, CA and SVM, despite achieving interesting results in anomaly detection, present some drawbacks when used separately. In the case of the CA, the network does not present uniform performance in defect detection, obtaining results well below its mean performance for some specific type of defects. On the other hand, the training of the one class SVMs presents some overfitting problems. By combining both approaches, these two problems become compensated. A good example of this effect can be observed in the detection of Defect 4 in the carpet texture. A poor initial detection result of 57.14% given by the CA is improved to 80.4% when the hybrid approach is carried out.

In the case of SVMs, overfitting problems can be observed in those defects detected with a 100% percentage, presented in Table 10. The compensation that the CA provides in these cases can be seen in the final detection results provided in Table 11 for those specific cases.

The values obtained after the combination, from the point of view of comparative analysis, serve to determine the best performance of the proposed approach with respect to the exclusive use of CA without SVM, and therefore, to determine that this method outperforms approaches such as the ones described in Section 1.2, where only CA is applied.

## 5. Conclusions

We designed a new method for general anomaly detection in textures by combining two different approaches that are complementary, CA and SVM.

We also paid special attention to the real manufacturing situations that occur in the industry, so that our solution was trained only with defect-free samples, modeling precisely the correct textures to detect possible failures and defects as outliers with respect to the learned ones.

The final results obtained for defect detection show an average value of 92% of correctly classified instances in all types of analyzed textures, which is a significant improvement over the existing approaches in the state of the art applied to these types of problems.

Another great advantage of this solution is that it does not require the intervention of experts to adjust its parameters. The learning of a new base texture to be analyzed for a new problem that could arise is automatic, provided that there is a database with a sufficient number of images for the precise training of the proposed algorithm. This last point does not present difficulties for the achievement of the same in the industrial facilities that deal with these types of quality control problems, overcoming also the natural setup problems of the supervised defect detection methods in real applications.

## Figures and Tables

**Table 1:** Reconstruction error obtained for both optimizers.

Optimizer	Number of Tile Texture Correct Images	Mean Reconstruction Error Obtained (%)
ADAM	1000	7.66
RMSprop	1000	5.25

**Table 2:** Defect free texture images in the dataset.

	Carpet	Grid	Leather	Tile	Wood
Number of images	279	263	244	229	246

**Table 3:** Number of images for each texture.

	Defects				
	Type 1	Type 2	Type 3	Type 4	Type 5
Carpet	18	16	16	16	18
Grid	11	11	10	10	10
Leather	18	18	16	18	17
Tile	16	17	15	17	14
Wood	7	9	9	20	10

**Table 4:** Typology of defects present in the different textures.

	Defect Type 1	Defect Type 2	Defect Type 3	Defect Type 4	Defect Type 5
Carpet	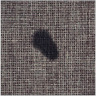	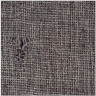	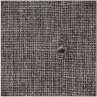	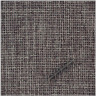	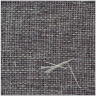
Grid	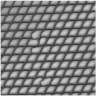	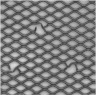	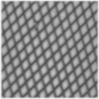	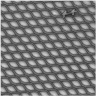	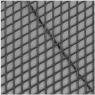
Leather	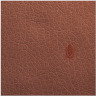	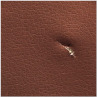	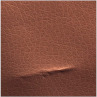	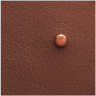	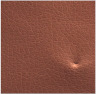
Tile	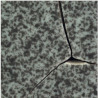	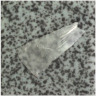	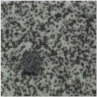	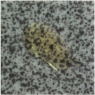	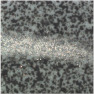
Wood	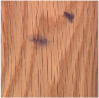	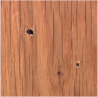	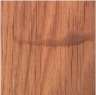	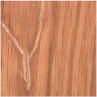	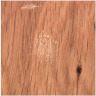

**Table 5:** Mean defect size in pixels for each texture.

Texture	Area (pix)
Carpet	13,256
Grid	3173
Leather	3655
Tile	61,587
Wood	9066

**Table 6:** Final number of images available in the modified dataset.

	Carpet	Grid	Leather	Tile	Wood
Correct images	6975	6575	6100	5725	6150
Defect 1	450	275	450	400	175
Defect 2	400	275	450	425	225
Defect 3	400	250	400	375	225
Defect 4	400	250	450	425	500
Defect 5	450	250	425	350	250

**Table 7:** Number of images in the modified and resized dataset for training and testing stages.

	Carpet	Grid	Leather	Tile	Wood
Training images without defects	4883	4603	4270	4008	4305
Testing images without defects	2092	1972	1830	1717	1845
Testing images with defects	2100	1300	2175	1975	1375

**Table 8:** Mean reconstruction error for all textures and their respective errors.

	Correct	Defect 1	Defect 2	Defect 3	Defect 4	Defect 5
Carpet	7.05	9.04	9.82	10.29	7.41	10.45
Grid	2.76	5.26	5.70	5.06	5.40	5.89
Leather	2.60	4.09	4.83	5.55	4.73	4.61
Tile	5.25	7.24	5.35	7.60	6.10	7.17
Wood	3.09	4.26	5.19	4.56	3.57	4.52

**Table 9:** CCP obtained with the convolutional autoencoder for all textures and errors.

	Defect 1	Defect 2	Defect 3	Defect 4	Defect 5	Mean
Carpet	78.37	90	100	57.14	70.21	79.144
Grid	95	85	93.75	86.66	84.21	88.924
Leather	100	68	91.66	58.62	90	81.656
Tile	86.59	55.78	83.87	78.88	69.38	74.9
Wood	84.93	88.23	90.59	72	84.44	84.038

**Table 10:** CCP results obtained with one class SVMs for all textures.

	Defect 1	Defect 2	Defect 3	Defect 4	Defect 5	Mean
Carpet	100	96.66	88.88	95.23	93.61	94.88
Grid	95	100	87.5	100	97.36	95.97
Leather	97.8	98.9	100	94	96.5	97.44
Tile	97.43	95.78	100	98.7	100	98.38
Wood	100	99.1	98.9	100	99	99.4

**Table 11:** Final results of texture error detection after hybridization of algorithms.

	Defect 1	Defect 2	Defect 3	Defect 4	Defect 5	Mean
Carpet	89.3	95.1	93.2	80.4	87.3	89.06
Grid	95	92.7	90	94.3	96.4	93.68
Leather	98.7	85.3	96.2	84.5	93.1	91.56
Tile	94.4	84.8	96.4	89	86.1	90.14
Wood	96.4	97.3	94.9	81.4	96.4	93.28

## Data Availability

Publicly available datasets were analyzed in this study. This data can be found here: [https://www.mvtec.com/company/research/datasets/mvtec-ad] (accessed on 5 April 2021).
